# Applications of the metaverse in medicine and healthcare

**DOI:** 10.1515/almed-2023-0124

**Published:** 2023-12-29

**Authors:** Tim Hulsen

**Affiliations:** Data Science & AI Engineering, Philips, Eindhoven, The Netherlands

**Keywords:** metaverse, telemedicine, digital twin, blockchain; medicine, healthcare

## Abstract

The metaverse is a virtual world that is being developed to allow people to interact with each other and with digital objects in a more immersive way. It involves the convergence of three major technological trends: telepresence, the digital twin, and blockchain. Telepresence is the ability of people to “be together” in a virtual way while not being close to each other. The digital twin is a virtual, digital equivalent of a patient, a medical device or even a hospital. Blockchain can be used by patients to keep their personal medical records secure. In medicine and healthcare, the metaverse could be used in several ways: (1) virtual medical consultations; (2) medical education and training; (3) patient education; (4) medical research; (5) drug development; (6) therapy and support; (7) laboratory medicine. The metaverse has the potential to enable more personalized, efficient, and accessible healthcare, improving patient outcomes and reducing healthcare costs. However, the implementation of the metaverse in medicine and healthcare will require careful consideration of ethical and privacy concerns, as well as social, technical and regulatory challenges. Overall, the future of the metaverse in healthcare looks bright, but new metaverse-specific laws should be created to help overcome any potential downsides.

## Introduction

**Figure 1:**
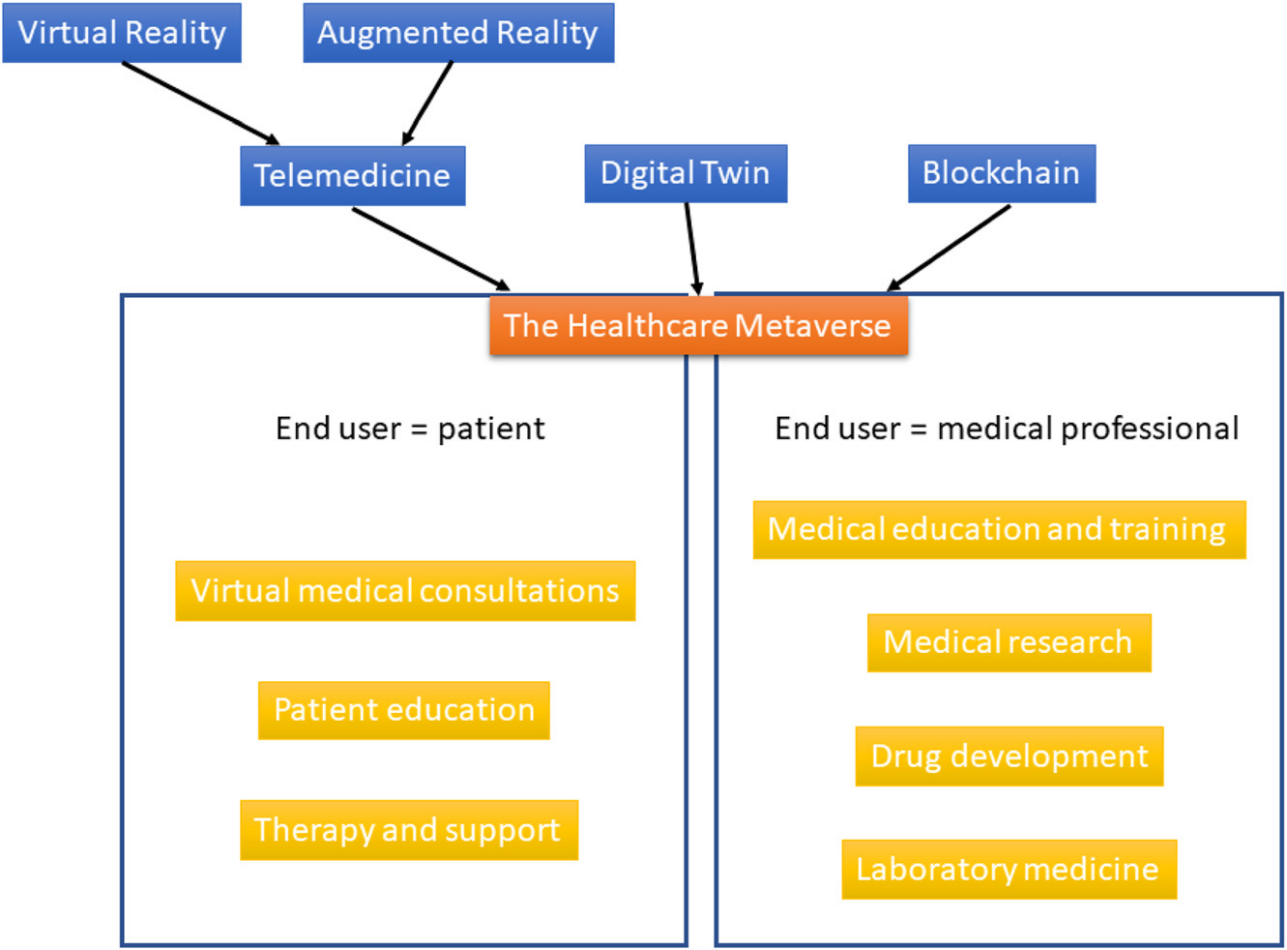
Components (blue) and application areas (yellow) of the healthcare metaverse.

The metaverse is an emerging technology that has the potential to transform many fields in the future. The metaverse is a virtual world that is being developed to allow people to interact with each other and with digital objects in a more immersive way than is currently possible through traditional screens and interfaces. It can be seen as a part of Web 3.0, in which we move to a decentralized web using technologies such as blockchain and artificial intelligence (AI) [1]. In the gaming world, the metaverse is already present [2] in, for example, gaming platforms such as Second Life, Minecraft and Roblox or Massively Multiplayer Online Role-Playing Games (MMORPGs) such as RuneScape or World of Warcraft. Other areas and industries are following the gaming world in setting up presence in the metaverse, such as tourism [3], marketing [4], transportation [5] and banking [6]. Facebook has even renamed its parent company to “Meta” in 2021, creating a new hype around the already existing metaverse [7]. A metaverse ecosystem, including an informal architecture, consisting of components in economy, ecology, technology and social, is presented in Figure 1 [8] of Faraboschi et al. [9]. Healthcare is now following this metaverse trend as well, although some of its applications are still in their infancy.

The metaverse involves the convergence of three major technological trends [10], which all have the potential to impact healthcare individually, but together they could create entirely new channels for delivering care that have the potential to lower costs and vastly improve patient outcomes (Figure 1). These are telepresence/telemedicine, the digital twin (DT) and blockchain. Telepresence is the ability of people to “be together” in a virtual way while not being close to each other. This can be achieved by virtual reality (VR, in which the user is completely immersed), augmented reality (AR, in which the user sees a real image combined with an artificial image), or other means. Besides VR and AR, Kye et al. [11] differentiates two other types of the metaverse: lifelogging (capturing, storing, and sharing everyday experiences and information about objects and people) and the mirror world (reflecting the real world as it is, but integrating and providing external environment information). Within healthcare, telepresence is mostly used in telemedicine, which is the provision of medicine as a remote service. Since the COVID-19 pandemic, the usage of telemedicine has grown at an exponential rate [12]. The DT is a virtual, digital equivalent to a physical product [13] and contains three main parts: the physical product, the virtual product and the connections of data and information that ties them together. In healthcare, the DT can be a virtual representation of a patient, a medical device or even a hospital. Blockchain is a type of Distributed Ledger Technology (DLT) mostly famous for being the underlying concept of cryptocurrency. It manages a record of transactions (not touching the original data) and additional data wrapped in several layers of data security (e.g., encryption). Blockchain plays two important roles in the metaverse: it serves as a repository to store data in the metaverse, and it can provide an economic system to connect the metaverse with the real world [14], for example, through the trading of non-fungible tokens (NFTs). In healthcare, blockchain is of specific interest, because it can provide a secure way of storing patient records and other sensitive data; a secure ‘wallet’ just like with cryptocurrency. Because of its decentralized setup, no single organization owns the data, giving control back to the patient. The use of blockchain in healthcare induces transparency and immutability [15]. In medicine and healthcare, the metaverse could be used in several ways, with the end user being either the patient or a medical professional (Figure 1): virtual medical consultations, medical education and training, patient education, medical research, drug development, therapy and support, and laboratory medicine.

Chengoden et al. [15] and Yang et al. [16] offer even more detailed lists of potential application areas. The metaverse has the potential to enable more personalized, efficient, and accessible healthcare, improving patient outcomes and reducing healthcare costs. However, as with any new technology, there are also potential risks and challenges that need to be addressed. In the following paragraphs we will have a look at each of the application areas, and we will discuss potential challenges as well as the future perspective [Figure 1].

## Application areas

### Virtual medical consultations

Virtual consultations, in which the doctor can talk to the patient using video conferencing technology such as Skype, Teams or Facetime, already exist for many years. However, when using these technologies, the patient might feel distant to the doctor. With VR, this distance feels smaller because it’s a much more immersive experience, enabling more personal discussions. Early VR prototypes to enable virtual medical consultations have existed since a few years, such as the Virtual Health Consultation [17] software. Zhang et al. [18] used mixed reality (MR) software for virtual consultations and other purposes, in which physical and virtual objects may co-exist and interact in real time. Kim et al. [19] developed a VR spatial psychological counselling service platform, in which counselling participants create and participate in their own avatars, and counselling facilitators respond in various virtual environments. Software like these could be part of a larger metaverse, allowing patients to receive medical advice and guidance without having to leave their homes. Virtual consultations save the doctor time and can be very useful for patients with reduced mobility. They could be integrated with DT, enabling the patient and the doctor to see a digital representation of the patient while discussing the patient’s health, improving the patient’s understanding of the disease.

### Medical education and training

The metaverse could be used to provide healthcare providers with virtual training and simulation experiences, allowing them to practice and improve their skills without putting patients at risk. Surgical simulations using VR were already proposed in the previous century [20]. Currently, several systems exist that make training and simulation in VR possible. For example, the Precision VR platform of Surgical Theater creates patient-specific 360°VR models from volumetric scans (e.g., CT, MRI) that can be manipulated and viewed from any angle using a touchscreen monitor or VR headset with controllers [21]. The MetaMedicsVR platform [22] provides VR training for any kind of healthcare professional, such as nurses, surgeons and other medical specialists. These educational VR systems have the key advantage that they can give immediate and personalized feedback to the student [23]. Sandrone [24] shows that metaverse games using avatars to undertake medical scenarios have successfully been piloted in many medical fields and in both preclinical and clinical medical education, improving learning outcomes and enhancing medical students’ engagement, participation and collaboration in a risk-free decision-making environment. This kind of ‘immersive learning’ technology is evolving rapidly and can transform medical education in the near future [25]. Recent studies show that many academic students nowadays prefer cyber-physical institutions to conventional institutions [26]. Besides training and simulation systems, there are also VR reference works. For example, the company Medical Augmented Intelligence (MAI) created “BodyMap” [27], a medically accurate representation of the human body that can be manipulated in 3D VR, to be used for medical education purposes.

### Patient education

The metaverse could be used to provide patients with virtual educational resources on topics such as healthy living and disease prevention [28]. They could also help the patient to get an impression of what their operation could look like, or what research has been carried out in the laboratory. “Pre-operative patient counseling services” aim to dispel patients’ fears by disclosing information related to anesthesia, surgical procedure, and post-surgical complications and by familiarizing them with the environment in which the operation will take place [29]. Advantages of virtual counseling over verbal counseling include: (1) reduced effect of a possible language barrier; (2) reduced time for the doctor and (3) no need for physically disabled patients to leave their home. The company HealthBlocks launched a blockchain-based app to assist users with getting active and staying healthy [30], and Healthify developed a sports and health metaverse based on blockchain technology [31]. DTs could be used to improve the understanding of a patient’s disease by showing a digital representation of the patient with the disease.

### Medical research

The metaverse could be used to simulate medical procedures and treatments, allowing researchers to test and refine new treatments and interventions. This is especially useful for treating mental disorders such as Attention Deficit Hyperactivity Disorder (ADHD) [32], Alzheimer’s disease (AD) [33] or diseases causing a reduced mobility, such as Parkinson’s disease (PD) [34]. In the latter case, VR interventions were carried out in the form of immersive games with individualized training programs, stimulating patients to work on their mobility as well as their autobiographical memory. VR rehabilitation training was proven to improve gait and balance of PD patients if used in combination with conventional rehabilitation training. Clinical trials could be sped up significantly by using the metaverse, breaking down physical and geographical barriers between clinicians and patients enrolled in a trial [35]. Zhang et al. [36] shows how the implementation of blockchain technology in clinical trials provides a secure and transparent platform for data management. Such a platform can promote the efficient and effective conduct of clinical trials, by improving the integrity and security of medical data, enhancing trust, and easing regulatory burden. Blockchain can also be used to improve the security of Electronic Health Records (EHRs) [37], making sure that researchers can use information from these medical records with reduced security risks. DTs can be used to simulate patients and medical devices, enabling researchers to perform studies without directly interfering with the patient [38]. Wang et al. [39] calls the healthcare metaverse “MeTAI” (“medical technology and AI”) and claims that it can facilitate the development, prototyping, evaluation, regulation, translation and refinement of AI-based medical practice, especially medical imaging-guided diagnosis and therapy.

### Drug development

The metaverse has the potential to accelerate drug development, improve the safety and efficacy of new drugs, and reduce the time and cost required to bring new drugs to market. The metaverse can enable the creation of virtual environments where drug developers can simulate the effects of drugs on the human body. These simulations can help researchers identify potential safety and efficacy issues with new drugs before they are tested in human clinical trials, potentially reducing the time and cost required for drug development. In drug design, VR can be used not only to visualize and interact with (DTs of the) molecules, but also to interact with molecular dynamics simulations ‘on the fly’ (also named “interactive molecular dynamics in VR” or “IMD-VR”) [40]. Examples of drug development software that support VR are UCSF ChimeraX [41] and YASARA [42], through which docking, the virtual simulation of molecular interactions, can take place. An example of IMD-VR software is Narupa iMD [43], which enables users to collaborate in a single virtual reality space and interact with real-time molecular simulations. Using these new techniques, pharmaceutical companies will be able to finish clinical studies in weeks rather than months or years, and at reduced cost [44]. Next to DTs of molecules, DTs of organs (e.g., the liver [45]) or model organisms (e.g., the mouse [46]) can be created to improve the drug development process. Design and recruitment of clinical trials, which are essential for testing the safety and efficacy of new drugs can be improved using the metaverse. Virtual simulations can be used to design more effective clinical trials, and virtual environments can be used to recruit and engage study participants more efficiently. It can also facilitate collaboration and data sharing among researchers, allowing them to pool their resources and expertise to develop new drugs more efficiently. Researchers from around the world can work together in virtual environments to share data, discuss research findings, and collaborate on drug development and bioinformatics [47] projects.

### Therapy and support

Patients suffering from certain phobias (e.g., agoraphobia [48], acrophobia [49], fear of flying [50]) or disorders such as posttraumatic stress disorder (PTSD) can be helped by applying ‘exposure therapy’ [51], in which they are exposed to situations that gives them an anxiety attack. Normally this would be done ‘*in vivo*’, in which the patient has to face his/her fear in real life, but it can also be done by providing a VR virtual environment which simulates the ‘dangerous’ situation. This VR exposure therapy (VRET) is more safe, convenient, controllable and cost-effective than its ‘*in vivo*’ counterpart. Recent developments in this field focus on making the VR experience more realistic, in order to create an ‘embodiment illusion’ using multisensory integrations in which patients feel as if they are really living in their virtual body (or ‘avatar’) [52]. This kind of ‘immersive virtual reality’ (IVR) shows promising results in people with mild to borderline intellectual disability (MBID) [53]. The metaverse could facilitate remote rehabilitation and physical therapy as well [54]. Patients could use VR exercises to improve their mobility and coordination from the comfort of their own home. This could be particularly useful for individuals living in rural or remote communities, or for those with mobility issues that make it difficult for them to travel to a healthcare facility. The metaverse could also be used to host virtual support groups and therapy sessions for patients with chronic conditions or mental health issues. These virtual support groups could provide a sense of community and connection for individuals who may not have access to in-person support.

### Laboratory medicine

Some of the uses of the metaverse described above, such as education and training, can also be applied in a medical laboratory setting, helping students, scientists and technicians to learn how to collaborate effectively in the laboratory. However, there are also some laboratory-specific types of use for all three components of the metaverse [55]. For example, DTs of laboratories and their equipment can be created [56], enabling smart manufacturing, in-silico modeling, pre-evaluation and simulation of assays and devices performances. Telepresence/VR can make possible dynamic 3D visits of virtual clinical laboratories and digital modeling of laboratory spaces, environments and workflows [57]. Another application is a blockchain-based remote laboratory management system [58], enabling secure data sharing between laboratory equipment as well as technicians and students. For bioinformatics and genomics research, there is software such as BioVR [59], which enables VR-assisted biological data integration and visualization. VR can be used for molecular modeling (see the section ‘Drug development’), multi-omics analysis, mesoscopic rigid body modelling or even 3D visualization of a virtual cell [60]. The blockchain is also used in bioinformatics, for example for the secure transmission of DNA sequencing data [61].

## Potential challenges

It is important to note that the implementation of the metaverse in medicine and healthcare will require careful consideration of ethical and privacy concerns, as well as social, technical and regulatory challenges. Ethical challenges include issues with integrity such as spreading false information and fraud, as well as intellectual property rights violations [62]. A digital environment such as the metaverse might also be susceptible to the advertisement of unhealthy products [63]. Just like with social media, the metaverse needs to have explicit moral guidelines to make sure that inappropriate behaviour will not be tolerated. Concerning privacy, hacking (causing the loss of sensitive personal information) might be difficult because of the inherent properties of the blockchain, but users are still rightfully concerned about their privacy in the metaverse [64]. Privacy laws from the real-world might not be accountable in the virtual domain [14], so metaverse-compatible privacy laws should be created with the collaboration of all stakeholders. Social challenges arise when people use the metaverse as a replacement of real, physical human contact, leading to loneliness. Moreover, when people with mental issues start using the metaverse, they might confuse the metaverse with reality, causing even more issues such as Cyber-Syndrome [65]. Technical issues are centered around ensuring the accuracy and reliability of virtual simulations. Especially for medical use, VR needs to be very reliable, which is often not the case at this moment [66]. VR/AR hardware is also still expensive and may cause side effects, including eye discomfort, strain, fatigue and blurred vision [24]. Finally, many of the challenges listed above have a regulatory aspect. Current cyberlaws are insufficient to deal with metaverse-specific problems. For example, who is liable and responsible in a virtual world? What if an avatar (or a virtual patient) commits a crime? If avatars are linked to a single ‘real’ person, then that person might be punishable, but what if avatars are partly steered by AI [67]? Perhaps avatars should be granted a separate legal personality [68], to keep avatars legally separated from humans. Just like with AI, where new laws are being created to deal with AI-specific issues [69], there need to be metaverse laws, created with the goal of providing a legal framework for ethics, privacy, social and technical aspects of the metaverse.

## Conclusions and outlook

Whereas the DT is a virtual representation of only a patient or a medical device, the metaverse offers a larger virtual environment with many possibilities. Current applications of the metaverse in healthcare are limited to, e.g., virtual trainings, therapy and support, and drug development. However, the future could be a virtual hospital, a virtual laboratory or even a virtual ‘care continuum’, the integrated system of care that guides and tracks patient over time through a comprehensive array of health services spanning all levels of intensity of care [70], both at home and in the clinic. The metaverse is very suitable to be used in healthcare, as it influences several key cognitive mechanisms: the experience of being in a place (e.g., a hospital or laboratory), the experience of being in a body (e.g., a virtual patient body), brain-to-brain individual attunement (e.g., doctor–patient interaction), brain-to-brain group synchrony (e.g., the virtual classroom), and emotions (e.g., emotional regulation during therapeutic sessions) (Table 1 of [71]). It also comes with several concerns in terms of ethics and privacy, as well as challenges in the social, technical and regulatory domains, which need to be overcome in order for the metaverse to be successful in healthcare. Overall, the future of the metaverse in healthcare looks bright, but just like with AI, new metaverse-specific laws and regulations should be created to help overcome any potential downsides.
